# Transcriptomics Analyses Reveal Wheat Responses to Drought Stress during Reproductive Stages under Field Conditions

**DOI:** 10.3389/fpls.2017.00592

**Published:** 2017-04-21

**Authors:** Jun Ma, Ruiqi Li, Hongguang Wang, Dongxiao Li, Xingyi Wang, Yuechen Zhang, Wenchao Zhen, Huijun Duan, Guijun Yan, Yanming Li

**Affiliations:** ^1^Faculty of Science, School of Plant Biology, The UWA Institute of Agriculture, The University of Western AustraliaPerth, WA, Australia; ^2^North China Key Laboratory for Crop Germplasm Resources of Education Ministry, College of Agronomy, Hebei Agricultural UniversityBaoding, China

**Keywords:** wheat, drought, RNA-seq, DEGs, developmental stages

## Abstract

Drought is a major abiotic stress that limits wheat production worldwide. To ensure food security for the rapidly increasing world population, improving wheat yield under drought stress is urgent and relevant. In this study, an RNA-seq analysis was conducted to study the effect of drought on wheat transcriptome changes during reproductive stages under field conditions. Our results indicated that drought stress during early reproductive periods had a more severe impact on wheat development, gene expression and yield than drought stress during flowering. In total, 115,656 wheat genes were detected, including 309 differentially expressed genes (DEGs) which responded to drought at various developmental stages. These DEGs were involved in many critical processes including floral development, photosynthetic activity and stomatal movement. At early developmental stages, the proteins of drought-responsive DEGs were mainly located in the nucleus, peroxisome, mitochondria, plasma membrane and chloroplast, indicating that these organelles play critical roles in drought tolerance in wheat. Furthermore, the validation of five DEGs confirmed their responsiveness to drought under different genetic backgrounds. Functional verification of DEGs of interest will occur in our subsequent research. Collectively, the results of this study not only advanced our understanding of wheat transcriptome changes under drought stress during early reproductive stages but also provided useful targets to manipulate drought tolerance in wheat at different development stages.

## Introduction

Drought is a major abiotic stress that limits wheat production worldwide. It can severely affect wheat growth and development causing various physiological and biochemical damage. Drought can lead to stomatal closure, reductions in photosynthesis and transpiration, growth inhibition, antioxidant production, and changes in hormonal composition (Szegletes et al., 2000; Lawlor and Cornic, 2002; Zhu, 2002). Depending on the onset time, duration and intensity of the stress, drought can cut wheat yields by up to 92% (Farooq et al., 2014). The IPCC forecasted that drought would be more frequent and severe in many crop-growing areas in the next decades due to climate change (IPCC, 2014). The world population is predicted to reach 9.6 billion by 2050, heightening the importance of improving drought tolerance in wheat to ensure food security for the rapidly growing population.

A survey of plant genes responsive to drought stress at the whole genome level is essential for understanding the biology of drought, and will, in turn, provide insight into the molecular mechanisms of drought (Hübner et al., 2015). In this regard, many drought-responsive wheat genes have been identified via microarray and RNA-seq technology (Aprile et al., 2009, 2013; Li et al., 2012; Liu et al., 2015). These genes responded to drought at different growth stages, including seedling, booting, anthesis and grain filling. These genes are a valuable genetic resource for drought tolerance improvement in wheat. By contrast, although drought during early reproductive stages (before booting) also impacted wheat development and yield potential (Oosterhuis and Cartwright, 1983; Qu, 1989; Ji et al., 2010), there have been no reported transcript study on the genes responsive to drought during these periods. Since many drought-related genes are genotype and growth stage specific (Garg et al., 2016), investigating wheat transcriptome changes under drought stress during early reproductive stages will help us to understand how drought affects gene expression during wheat development.

In the current study, we performed a transcriptomic analysis of a drought-tolerant winter wheat variety “Luyuan502” subjected to drought stress at various growth stages. Because gene expression can change under different environments (Xu, 2016), the plants in our study were grown under field conditions to simulate actual farming practices rather than using growth chambers like previous studies. DEGs at five different time points during early and late reproductive periods were investigated for biological function. Furthermore, we selected five DEGs for RT-qPCR validation using a drought-tolerant and a drought-sensitive variety.

## Materials and methods

### Plant materials and stress treatments

**Figure 1:**
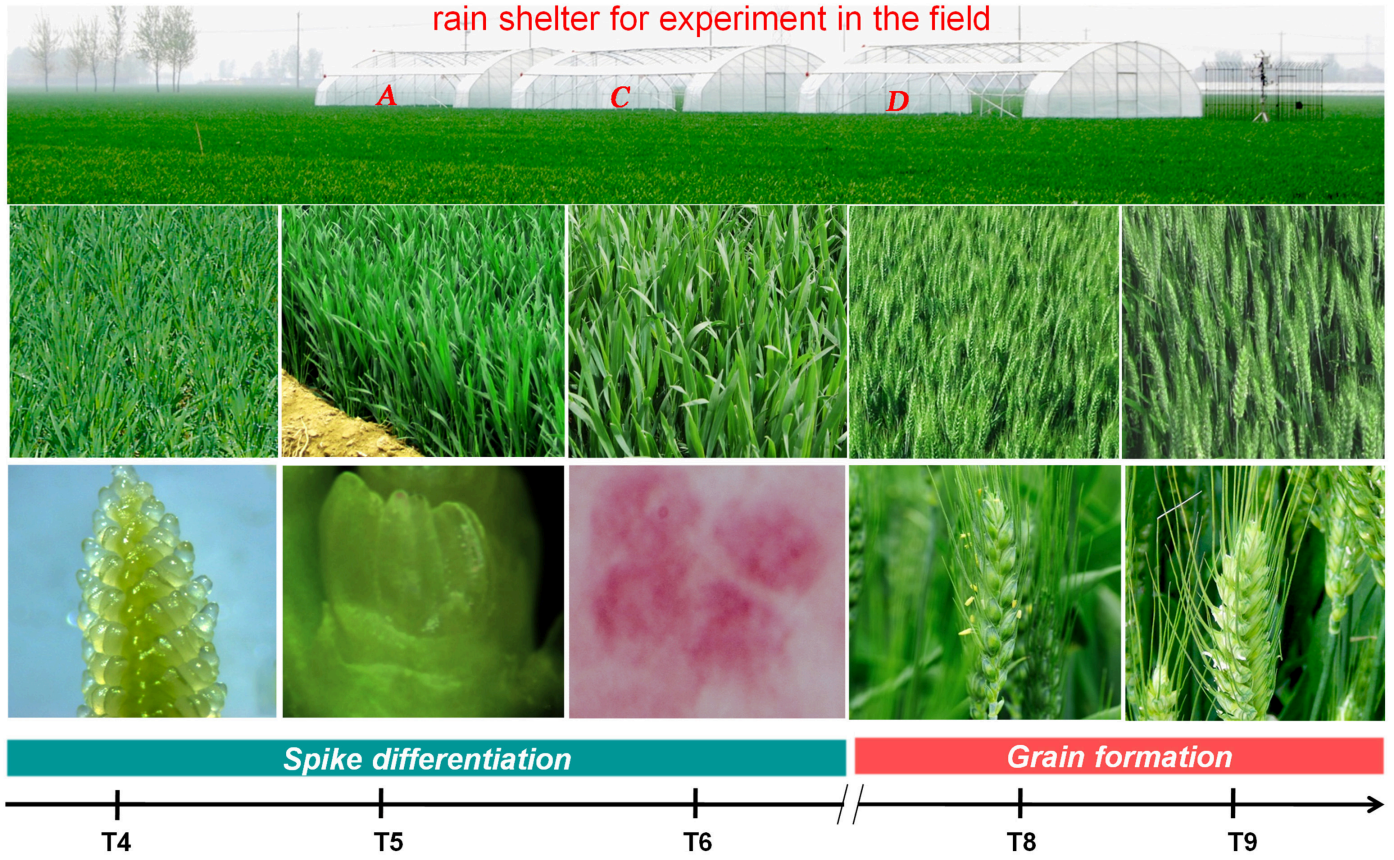
**The developmental stages of plants used for tissue collection and RNA-seq analysis**. T4: pistil and stamen differentiation stage; T5: anther differentiation stage; T6: tetrad stage; T8: early flowering stage; and T9: grain formation stage.

**Figure 2:**
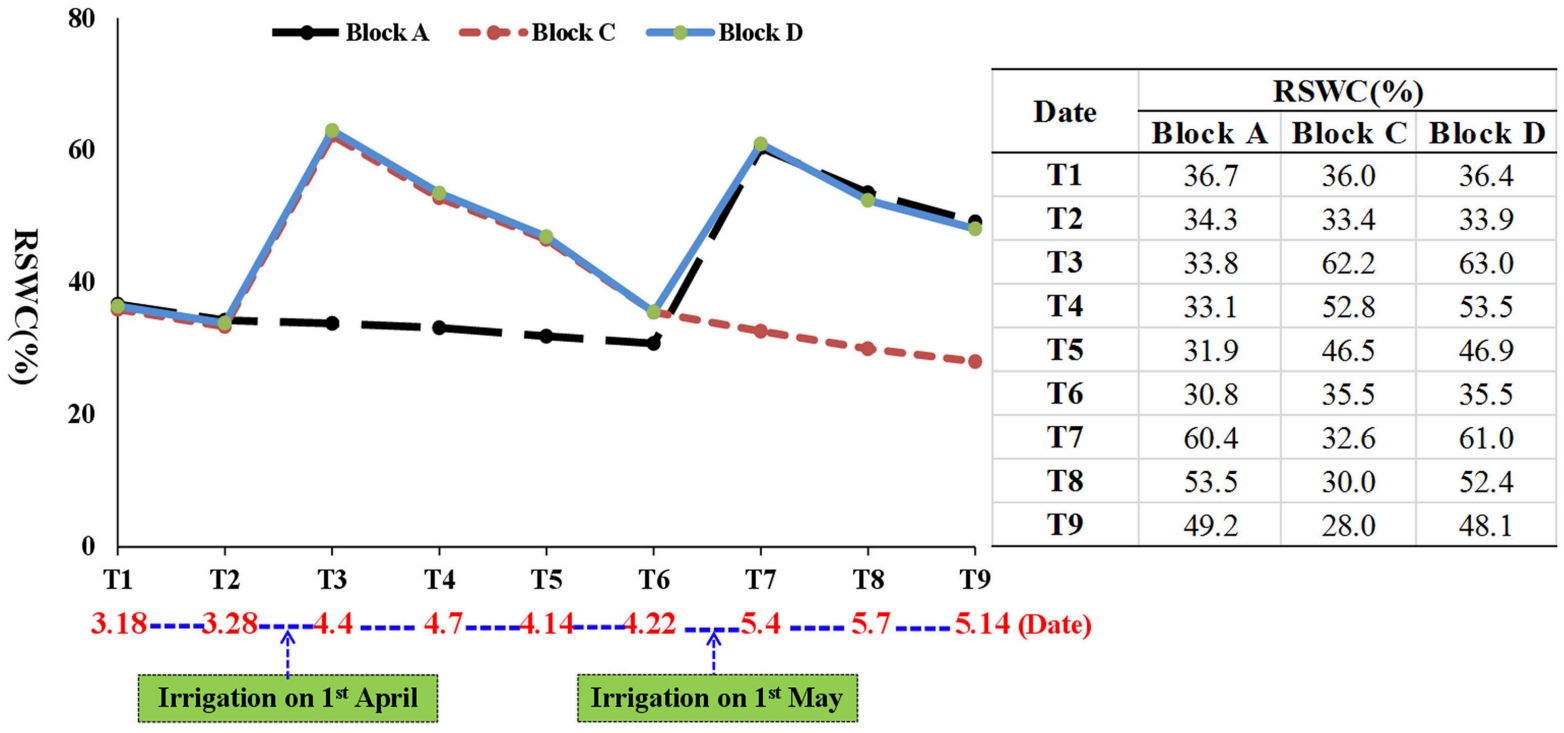
**Profile of relative soil water content (RSWC) during wheat growth**. T: time point.

Based on a survey of 156 elite commercial wheat varieties in the North China Plain, we selected Luyuan 502 for its drought tolerance (Li, 2014). Seeds of Luyuan 502 were field sown in early October 2014 at the Experimental Station of the Hebei Agricultural University in Gaocheng County, China (114.84° E 38.03° N, 56 m above sea level). Plants were arranged in three blocks (A, C, and D), each being 12 m long and 8.5 m wide, with a rain shelter to keep the plants from rain and snow (Figure 1). To simulate local farming practices, a two-time irrigation strategy was adopted. Block D (control block) was watered twice, whereas block A and block C was watered once. On 1 April 2015, when the first internodes of most plants (>80%) were visible (Feekes' growth stage 6), the plants in blocks C and D were watered. On 1 May 2015, when most of the plants (>80%) had begun to flower (Feekes' growth stage 10.5.1) (Large, 1954), plants in blocks A and D were watered. About 574 kg of water (equal to 60 mm) was used for each irrigation. During wheat growth, the relative soil water content (RSWC) was recorded at nine time points (T1 to T9) to monitor the level of drought stress in the three blocks (Figure 2). The RSWC was calculated as RSWC = [(current pot weight − soil dry weight)/weight of soil watered to field capacity] × 100.

Plant samples were taken in blocks A and D at T4 (7 April; 7 days after first irrigation), T5 (14 April; 14 days after first irrigation) and T6 (21 April; 21 days after first irrigation), and in blocks C and D at T8 (7 May; 7 days after second irrigation), and T9 (14 May; 14 days after second irrigation) (Figure 2). At T4, T5, and T6, the upper parts of main tillers were collected, while at T8 and T9, the developing spikes were collected after removing awns and rachis. The developmental stages of plants at these five time points were checked by microscope or the naked eye. Plant materials from five individual plants in each block were pooled as a biological replicate for RNA extraction. Three biological replicates were collected at each time point, resulting in 30 samples for cDNA library construction and sequencing (three biological replicates × two blocks × five time points = 30 samples). The collected samples were frozen immediately in liquid nitrogen and stored at −80°C until required. To examine the effects of drought on plant growth and development in each block, total dry biomass before flowering, total dry biomass at maturity, spike and spikelet numbers, number of fertile and infertile spikelets per spike, thousand grain weight and grain yield were recorded. The differences between grain setting rate and grain yield difference to the control were also calculated.

### RNA isolation, library construction, transcriptome sequencing, and reads mapping

Total RNA was extracted using TRIzol reagent (Invitrogen) according to the manufacturer's instructions. DNA was removed by digestion with RNase-free DNase (Qiagen), and RNA was purified and concentrated using an RNeasy column (Qiagen). RNA quality was evaluated by gel electrophoresis, spectrophotometer analysis and an Agilent 2100 bioanalyzer. RNA samples were sent to Novogene Bioinformatics Technology Co. Ltd., Beijing, China (http://www.novogene.cn) for further processing and sequencing. The sequencing libraries were sequenced on Illumina HiSeq 2500 (Illumina, San Diego, USA) and 125/150 bp paired-end reads were generated. The RNA sequences have been deposited at the National Center for Biotechnology Information (NCBI) with the accession number of SRP102636. The raw reads were filtered by removing adapter sequences, ambiguous nucleotides (if the proportion of “Ns” exceeded 10%), and low-quality sequences (when the percentage of bases with low Phred quality score ≤20 is greater than 50% in a read). At the same time, Q20, Q30, and GC contents of the clean data were calculated. All downstream analyses were based on clean data.

The clean reads were aligned to wheat reference genome sequences released by the International Wheat Genome Sequencing Consortium (IWGSC) (ftp://ftp.ensemblgenomes.org/pub/release-23/plants/fasta/triticum_aestivum/dna/) using TopHat 2.0.12 (Trapnell et al., 2010). Mismatches of no more than two bases were allowed in the alignment. After alignment, HTSeq v0.6.1 was used to count the read numbers that were mapped to each gene. The expected number of Fragments Per Kilobase of transcript sequence per Million base pairs sequence (FPKM) of each gene was calculated to determine the expression values of this gene. Pearson's correlation coefficients between the three biological replicates were calculated in the R software package to quantify the correlation between biological replicates.

### DEGs identification and functional annotation

A consensus FPKM across the three biological replicates was calculated and used in the DEG analysis (Trapnell et al., 2010). For each gene, the relative homologous rice gene, homologous rice gene annotation, and NCBI non-redundant protein database annotation were investigated. DEGs were identified using DESeq 1.10.1 (Anders and Huber, 2010). To assess the variability among samples, we performed principal component analysis (PCA) for the wheat genes identified at T4, T5, and T6 using the prcomp command with default parameters in the R software package (Robinson et al., 2010).

Five pair-wise comparisons between the three blocks were made to identify the drought-responsive genes. At T4, T5, and T6, the comparison was made between blocks A and D while at T8 and T9, the comparison was made between blocks C and D. A corrected *p-*value < 0.05 was set as the criteria for determining DEGs. We created heatmaps to demonstrate the gene expression data of T4 using Java TreeView (Robinson et al., 2010). The subcellular locations of proteins for the DEGs at T4 were analyzed using PSORT Prediction software (http://psort.hgc.jp/form.html).

To study the biological significance of the DEGs, an enrichment analysis of gene ontology (GO) terms was conducted with GO seq R package. Statistically significant over-representation of GO categories (corrected *p*-value < 0.05) in response to the drought treatment was determined separately for each time point. Similarly, KOBAS software was used to test the statistically enriched pathways associated with the DEGs at each time point in the KEGG (Kyoto Encyclopedia of Genes and Genomes) database.

### Validation of RNA-Seq analysis by RT-qPCR

To confirm the RNA-seq results, among the identified DEGs in Luyuan 502, 21 genes were randomly selected and assessed using RT-qPCR. RT-qPCRs were conducted on a StepOne Plus Real-Time PCR system (Applied Biosystems) using SYBR Green I for the detection of PCR products. Each reaction was performed in a final volume of 16 μL, containing 8 μL SYBR Green PCR Master Mix (Applied Biosystems), 250 nM of each primer and 50 ng cDNA template. The thermal cycling conditions were 94°C for 10 min, followed by 40 cycles of 94°C for 15 s, 55°C for 30 s, and 60°C for 1 min, with fluorescence detection at the end of each cycle. The amplification of a single product per reaction was confirmed by melting curve analysis. All reactions were performed in three technical triplicates. Wheat α*-tubulin* (forward: ATCTGTGCCTTGACCGTATCAGG; reverse: GACATCAACATTCAGGACACCATC) was used as an internal reference gene to normalize Ct values of each reaction (Chen et al., 2016), which were determined using the CFX96 software with default settings. The sequences of the 21 genes and primers used in the RT-qPCR analysis are listed in Table S5.

### Validation of DEGs at early reproductive stages under different genetic backgrounds

A drought-tolerant variety Cangmai 6001 and a drought-sensitive variety Hanmai 9 were subjected to normal watering as a control, and 5 days of non-watering as a drought treatment until the RSWC reached the threshold (35%) at the tilling stage in the glasshouse. To confirm their different sensitivities to drought, four drought-related physiological indexes—ascorbate peroxidases (APX) activity, catalase (CAT) activity, H_2_O_2_ content and MDA content—were measured in both varieties before sampling.

Wheat leaves subjected to drought stress or mock treatments were sampled for physiological index determination. The MDA (malondialdehyde) level was estimated according to Li et al. (2014). H_2_O_2_ (hydrogen peroxide) accumulation was assessed using commercial kits (Jiancheng Biotech Inc., Nanjing, China) according to Yang et al. (2016). Each sample was homogenized in pre-cooled phosphate-buffered saline (PBS) using 1 mL of buffer per 0.1 g of fresh tissue. The homogenate was centrifuged at 10,000 g for 10 min at 4°C. Freshly isolated supernatant fractions were used immediately for measuring H_2_O_2_ content. Adduct formation was measured spectrophotometrically at 405 nm using Thermo Scientific Multiskan FC (Shanghai, China) in strict accordance with the manufacturer's instructions. Protein contents were determined using an Enhanced BCA Protein Assay Kit (Beyotime, Shanghai, China). The activities of antioxidant enzymes, including catalase (CAT) and ascorbate peroxidase (APX), were measured as described previously (Rao et al., 1996; Li et al., 2011; Tian et al., 2014). The assay of enzyme activity was carried out using a spectrophotometer at 25°C.

As we mainly focused on genes differentially expressed during early reproductive stages, we selected five DEGs during early reproductive stages for RT-qPCR validation: Traes_5DS_CCCDA48421 (T4), Traes_5BS_9584239E51 (T4), Traes_2DL_77F25CE27 (T4 & T5), Traes_3DL_304C8DD67 (T4 & T5) and Traes_7DS_1D74598FD (T4 & T6). These five DEGs were associated with floral organ development, stomatal movement or photosynthesis activity etc. RNA of Hanmai 9 and Cangmai 6001 was extracted using TRIzol reagent (Invitrogen). Three trials were conducted for the measurement of four drought-related physiological indexes and RT-qPCR analysis. A general mean across each trial was calculated and used. Two-tailed unpaired Student's *t*-tests was used to determine if the differences between the two varieties were significant or not. Wheat α*-tubulin* was used as the reference gene (Chen et al., 2016). RT-qPCR primer sequences are listed in Table S5.

## Results

### RSWC and plant response to drought

Initially, RSWC in blocks A, C, and D was similar at T1 and T2. The first irrigation on 1 April in blocks C and D, increased RSWC in these two blocks to 62.2 and 63.0% at T3, then decreased to 52.8 and 53.5% at T4, 46.5 and 46.9% at T5, and 35.5% at T6. In contrast, RSWC in the unirrigated block A changed little (36.7–30.8%) during the same period. The second irrigation on 1 May in blocks A and D increased RSWC in these two blocks to a much higher level than in block C at T7, T8 and T9 (Figure 2).

Total plant biomass was a direct growth parameter to measure the effects of drought on wheat growth and development. As expected, the plants in block A which suffered drought stress during early reproductive stages had the lowest total dry biomass before flowering. At maturity, due to second irrigation, the biomass of plants in block A had increased to a similar level as that in block C, but was still much lower than block D (control). A similar trend was observed for grain setting rate and yield. Blocks A and C had lower values (−6.6 and −6.1%; −4.9 and −2.4%) on these two important traits compared with block D. Detailed information on the traits measured in the three blocks is shown in Table 1.

**Table 1:** **Yield related traits in each block at harvesting**.

**Block**	**TDB1**	**TDB2**	**SN**	**SLN**	**NFS**	**NIS**	**GSRD**	**TGW**	**GY**	**GYD**
A	12412.3a	19294.5a	696a	18.2a	15.4a	2.8b	−6.6%	46.0b	8946.5b	−6.1%
C	13695.1b	19307.1a	750b	18.0a	15.5a	2.5b	−4.9%	41.7a	9294.5a	−2.4%
D	13695.5b	19963.5a	744b	18.4a	16.7b	1.7a	0	44.5b	9522.9a	0

*TDB1, total dry biomass before flowering (kg/hm^2^); TDB2, total dry biomass at maturity (kg/hm^2^); SN, spike number (104/hm^2^); SLN, spikelet number; NFS, number of fertile spikelets per spike; NIS, number of infertile spikelets per spike; GSRD, grain setting rate difference to control; TGW, thousand grain weight (g); GY, grain yield (kg/hm^2^); GYD, grain yield difference to control. The student's t-test was performed for statistical analysis, and different letters within a column indicate statistically significant differences between blocks at p < 0.01*.

Microscopic inspection determined that plants at T4, T5, and T6 were at the pistil and stamen differentiation stage, anther differentiation stage and tetrad stage, respectively. Plants at T8 and T9 were at the early flowering and grain formation stages, respectively (Figure 1).

### Summary of RNA-Seq data

**Figure 3:**
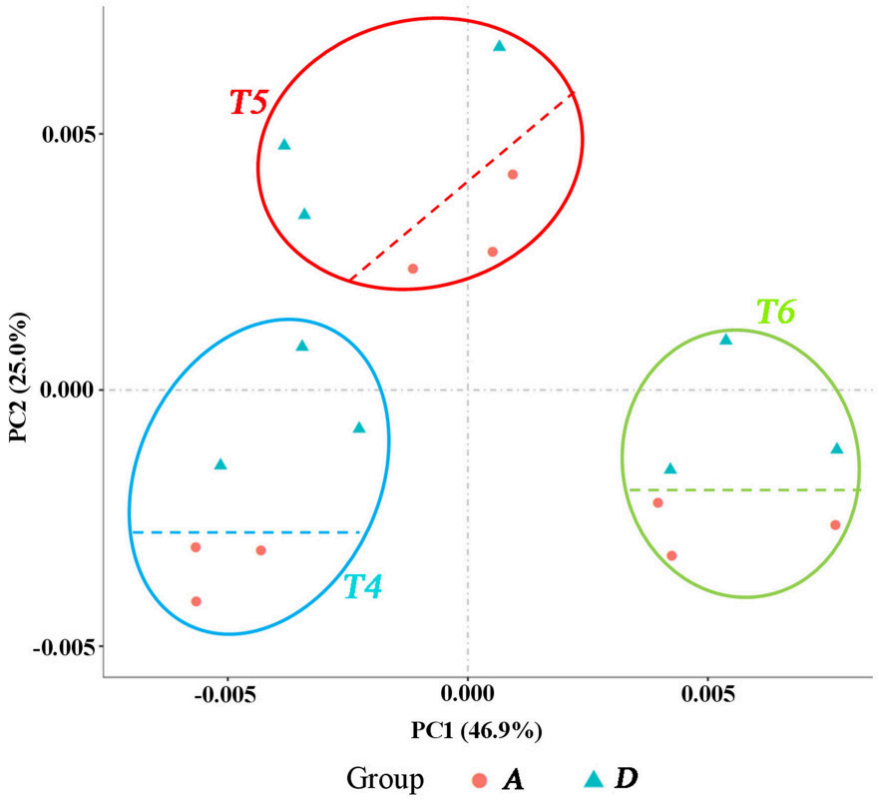
**PCA displaying the intrinsic biological variation among samples**. Group A: samples from block A. Group D: samples from Block D. The three trilaterals or roundness in each ellipse represents three biological replicates at the same time point (development stage).

The quality of total RNA was good (Figure S1). Of the 1.7 billion raw reads generated from the 30 cDNA libraries, approximately 1.6 billion clean reads were identified, ranging from 47 million to 69 million reads per library. Clean reads were mapped to the wheat genome released by IWGSC using TopHat 2.0.11. The mapping rates of each library ranged from 98.74 to 99.14%. A total of 99,411 genes from IWGSC and 16,244 novel isoforms of known genes were identified (Table 2). The genes with FPKM (transcript abundance of the gene) above 1.0 accounted for around 40%, and those above 3.0 accounted for around 25%. The biological replicates were highly correlated. The Pearson's correlation coefficients among the three replicates for each time point ranged from 0.82 to 0.98 (Figure S2), indicating that the three replicates were consistant. The PCA analysis indicated that most of the variation in gene expression among different ellipses was a consequence of the developmental stage. Furthermore, two distinct groups formed within each ellipse; one comprised plants in block A and the other comprised plants in block D, indicating that the transcriptomes of drought and irrigated plants clearly differed from each other (Figure 3).

**Table 2:** **Number of reads sequenced and mapped with Tophat at each time point**.

**Time point**	**Block**	**Raw reads**	**Clean reads**	**Total mapped reads**	**Reads mapped to gene (%)**
T4	A	55,101,326	52,359,821	39,568,121	99.00
	C	66,209,493	63,131,921	48,451,375	98.97
T5	A	53,179,667	51,277,891	39,809,444	98.90
	C	60,931,653	58,045,403	45,280,010	99.07
T6	A	52,067,162	50,169,858	38,980,092	98.97
	C	64,661,216	61,811,627	48,822,803	98.94
T8	C	56,984,949	54,509,265	42,577,251	98.74
	D	54,114,718	52,046,468	40,899,491	98.80
T9	C	51,186,386	48,646,447	38,516,348	98.83
	D	52,790,373	50,615,959	39,526,817	99.14

*The average value of three biological replicates for each stage was calculated and used in the table*.

### DEGs, functional annotation of DEGs and KEGG pathway analysis

Drought induced 309 DEGs across different time points: 226, 44, 8, 6, and 25 DEGs at T4, T5, T6, T8, and T9, respectively (**Figure 5**, Table S1). The GO terms and KEGG pathways associated with those DEGs are listed in Tables S2, S3, respectively.

**Figure 4:**
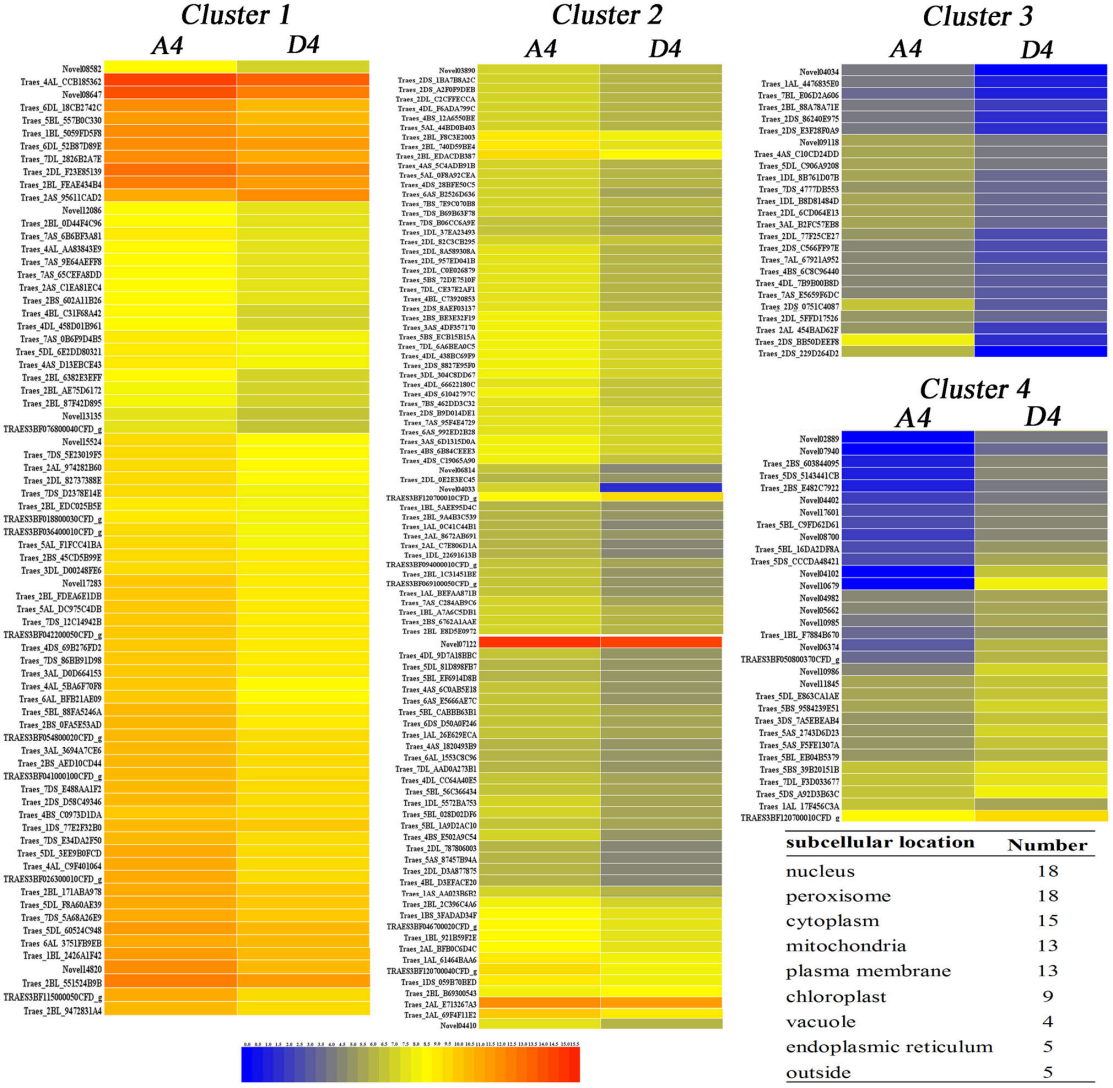
**Heatmap of 226 drought-responsive genes at T4 (pistil and stamen differentiation stage) based on fpkm values for each gene**. The color scale of blue (low), yellow (medium), and red (high) represents the transcriptome levels of differentially expressed genes. The predicted subcellular location of 100 proteins from 226 genes was located mainly in the plasma membrane, vacuole and endoplasmic reticulum.

At the pistil and stamen differentiation stage (T4), 193 up-regulated DEGs and 33 down-regulated DEGs were identified. Their expression patterns are shown in Figure 4. The genes in clusters 1–4 were up-regulated genes by drought, whereas those in cluster 5 were down-regulated genes. Furthermore, subcellular location analysis of 100 proteins of the DEGs revealed that they were mainly located in the nucleus (18), peroxisomes (18), mitochondria (13), plasma membrane (13), and chloroplasts (9) (Figure 4 and Table S4). These results suggest that these organelles play critical roles in drought stress, and that the peroxisome, nucleus and mitochondria may be more sensitive to drought stress damage in wheat at early developmental stages. GO analysis showed that the “protein disulfide oxidoreductase activity” and “cell redox homeostasis” were enriched (Table 3). Four KEGG pathways-“galactose metabolism,” “circadian rhythm–plant,” “starch and sucrose metabolism” and “flavonoid biosynthesis”-were significantly impacted by drought stress (Table 4).

**Table 3:** **GO terms significantly enriched for the DEGs at each time point**.

**Time**	**GO term**	**Corrected *p*-value**	**Gene**
T4 - pistil and stamen differentiation stage	Protein disulfide oxidoreductase activity	<0.01	TRAES3BF036400010CFD_g
			Traes_5AS_F5FE1307A
			Traes_5AS_2743D6D23
			Traes_5BS_9584239E5
			Traes_5BS_39B20151B
			Traes_5DS_A92D3B63C
			Traes_5DS_5143441CB
			Traes_5DS_CCCDA48421
	Cell redox homeostasis	<0.01	TRAES3BF036400010CFD_g
			Traes_5AS_F5FE1307A
			Traes_5AS_2743D6D23
			Traes_5BS_9584239E5
			Traes_5BS_39B20151B
			Traes_5DS_A92D3B63C
			Traes_5DS_5143441CB
			Traes_5DS_CCCDA48421
T5 - anther differentiation stage	Magnesium ion binding	<0.01	TRAES3BF007300100CFD_g
			TRAES3BF007300110CFD_g
			TRAES3BF136800060CFD_g
			Traes_5DL_CC4E32ECC
			Traes_7AS_97A8A5101
			Traes_7DS_1D74598FD
	Ribulose-bisphosphate carboxylase activity	<0.01	TRAES3BF007300100CFD_g
			Traes_7AS_97A8A5101
			Traes_7DS_1D74598FD
	Carbon fixation	<0.01	TRAES3BF007300100CFD_g
			Traes_7DS_1D74598FD
			Traes_7AS_97A8A5101
T9 - grain formation stage	Alpha-amylase activity	<0.01	Traes_5BL_D1DF6C31E
			Traes_5BL_AC845F1C1
			Traes_5DL_91B56C21D
	Calcium ion binding	<0.05	Traes_5AL_3D9E58850
			Traes_5BL_AC845F1C1
			Traes_5BL_D1DF6C31E
			Traes_5DL_91B56C21D

**Table 4:** **KEGG pathways significantly enriched for the DEGs at each time point**.

**Time point**	**KEGG pathway**	**Corrected *p*-value**	**Gene**
T4 - pistil and stamen differentiation stage	Galactose metabolism	<0.01	Traes_7DS_E488AA1F2|Traes_7AS_6B6BF3A81|
			Traes_7DS_5A68A26E9|Traes_7DS_E34DA2F50|
			Traes_5DL_E863CA1AE|Traes_4AL_C9F401064|
			Traes_7DS_5E23019F5|Novel13135
	Circadian rhythm - plant	<0.01	Traes_2BS_0FA5E53AD|Traes_2DS_B9D014DE1|
			Traes_7DS_12C14942B|Traes_2DS_1BA7B8A2C|
			Traes_3AS_6D1315D0A|Traes_2DS_8827E95F0
	Flavonoid biosynthesis	<0.01	Traes_2DS_1BA7B8A2C|Traes_1BL_2426A1F42|
			Traes_2DS_8827E95F0|Traes_2BS_0FA5E53AD|
			Traes_2DS_B9D014DE1
	Starch and sucrose metabolism	<0.05	Traes_7DS_E488AA1F2|Traes_7AS_6B6BF3A81|
			Traes_7DS_5A68A26E9|Traes_7DS_E34DA2F50|
			Traes_4AL_C9F401064|Traes_7DS_5E23019F5|
			Novel13135
T5 - anther differentiation stage	Carbon fixation in photosynthetic organisms	<0.01	Novel10984|Traes_7DS_1D74598FD|
			TRAES3BF007300110CFD_g|Traes_7AS_97A8A5101|
			TRAES3BF007300100CFD_g|Traes_5DL_CC4E32ECC|
			TRAES3BF136800060CFD_g
	Glyoxylate and dicarboxylate metabolism	<0.01	Traes_7DS_1D74598FD|TRAES3BF007300110CFD_g|
			Traes_7AS_97A8A5101|TRAES3BF007300100CFD_g|
			Traes_5DL_CC4E32ECC|TRAES3BF136800060CFD_g
	Carbon metabolism	<0.01	Novel10984|Traes_7DS_1D74598FD|
			TRAES3BF007300110CFD_g|Traes_7AS_97A8A5101|
			TRAES3BF007300100CFD_g|Traes_5DL_CC4E32ECC|
			TRAES3BF136800060CFD_g
T6 - tetrad stage	Porphyrin and chlorophyll metabolism	<0.05	Traes_2DS_BB50DEEF8
T9 - grain formation stage	Starch and sucrose metabolism	<0.01	Traes_5BL_AC845F1C1|Traes_5DL_91B56C21D|
			Traes_5BL_D1DF6C31E|Traes_2BL_33E5CFD18

**Figure 5:**
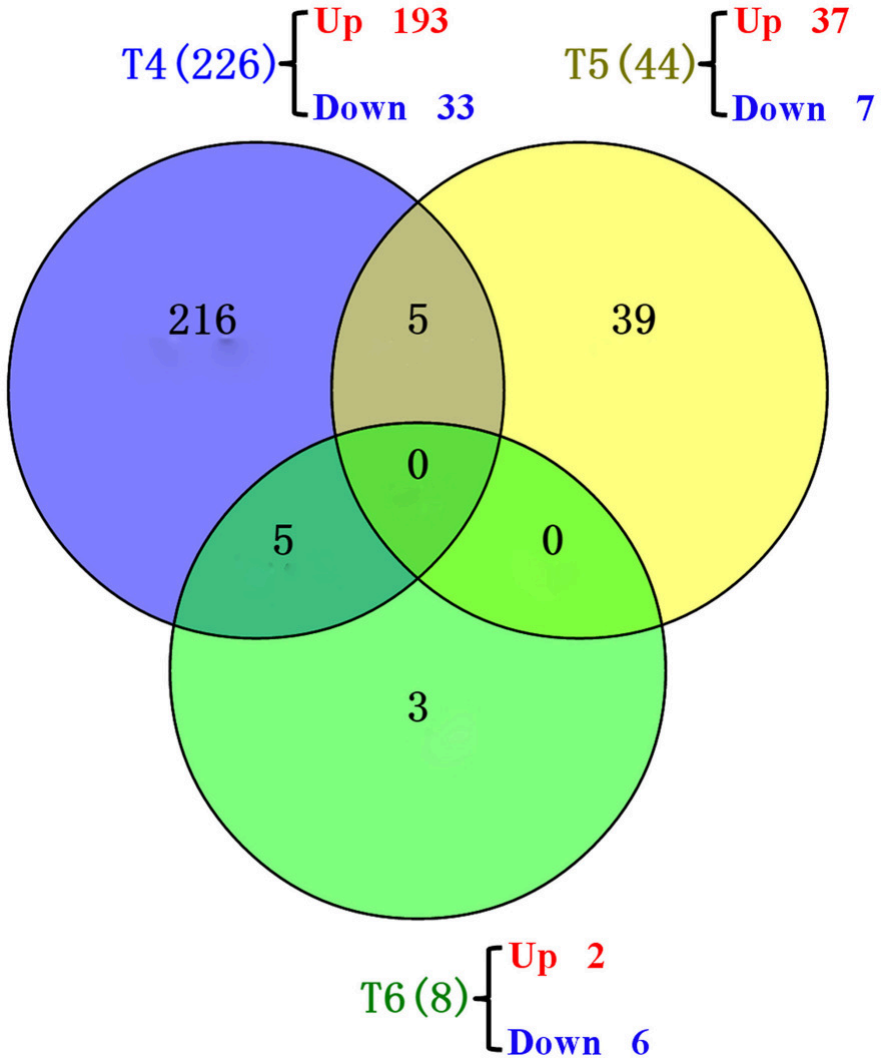
**Venn diagrams showing the common genes at different time points**. T4 (blue): pistil and stamen differentiation stage; T5 (yellow): anther differentiation stage; T6 (green): tetrad stage.

At the anther differentiation stage (T5), 37 up-regulated DEGs and seven down-regulated DEGs were identified. Three GO terms “magnesium ion binding,” “carbon fixation,” and “ribulose-bisphosphate carboxylase activity” were over-represented (Table 3). The “carbon fixation in photosynthetic organisms,” “glyoxylate and dicarboxylate metabolism” and “carbon metabolism” pathways were significantly enriched (Table 4). Interestingly, five DEGs were shared between the pistil and stamen differentiation stage (T4) and the anther differentiation stage (T5) (Figure 5). Of these, the drought up-regulated DEG Traes_3DL_304C8DD67 was annotated to the vacuolar-processing enzyme (VPE), which has been associated with growth inhibition, cell death and stomatal movement in *Arabidopsis* (Hara-Nishimura et al., 2005; Yamada et al., 2005; Albertini et al., 2014).

At the tetrad stage (T6), two up-regulated DEGs and six down-regulated DEGs were identified. The pathways of metabolism of porphyrin and chlorophyll were significantly affected (Table 4). This enriched pathway only contains one DEG, Traes_2DS_BB50DEEF8, which was homologous to the rice gene LOC_Os03g20700 encoding magnesium chelatase H subunit (CHLH). In our study, the expression of Traes_2DS_BB50DEEF8 in the water-stressed block A was 4.53-fold higher than that in the control block D, suggesting a positive response of this gene to drought stress. Traes_2DS_BB50DEEF8 was one of the five genes shared between T4 and T6 (Figure 5). The CHLH subunit affected plant hormone abscisic acid (ABA) signaling in stomatal guard cells, and overexpression of the CHLH gene in guard cells improved plant drought tolerance by promoting stomatal closure (Tsuzuki et al., 2013).

Compared with earlier reproductive periods, the number of DEGs detected during flowering declined. During early flowering (T8), six DEGs were identified, including one up-regulated gene and five down-regulated genes. The down-regulated gene TRAES3BF077200010CFD_g encoded LTPL38 which may contribute to mineral accumulation in wheat grain (Singh et al., 2014). At the grain formation stage (T9), 25 DEGs were identified (two up-regulated and 23 down-regulated). The GO terms “alpha-amylase activity” and “calcium ion binding” were enriched (Table 3). The “starch and sucrose metabolism” pathway was enriched (Table 4).

### Validation of DEGs through RT-qPCR

**Figure 6:**
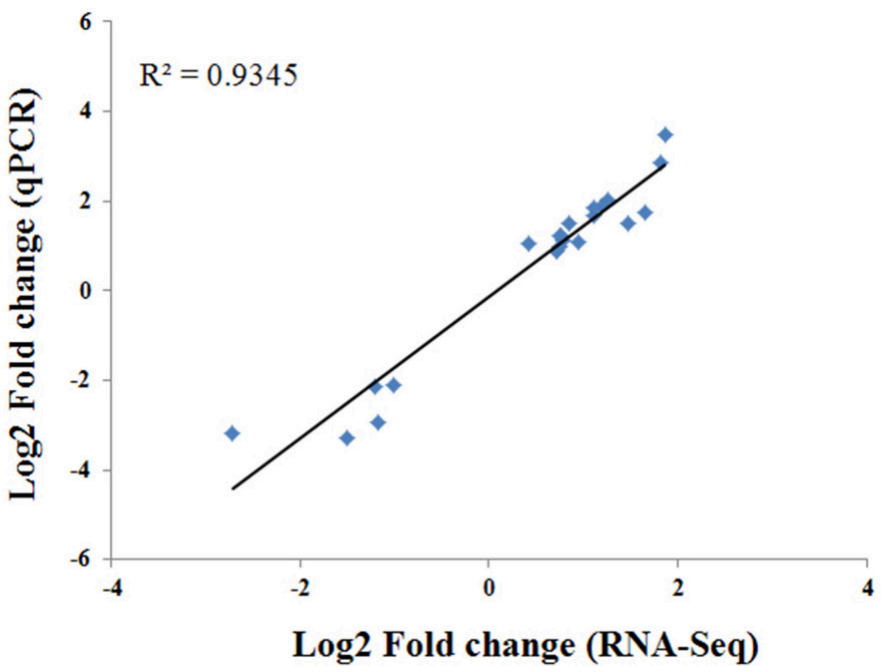
**Comparison of the log2 fold change of 21 selected transcripts using RNA-seq and RT-qPCR**.

To verify the RNA-seq results, RT-qPCR analysis was conducted against 21 randomly selected genes. The expression patterns of these 21 genes assessed by RT-qPCR correlated well (*R*^2^ = 0.9345) with those obtained from the RNA-seq analysis (Figure 6). These results confirm the accuracy of the transcriptome changes obtained by RNA-seq in this study.

### Validation of DEGs under different genetic backgrounds

**Figure 7:**
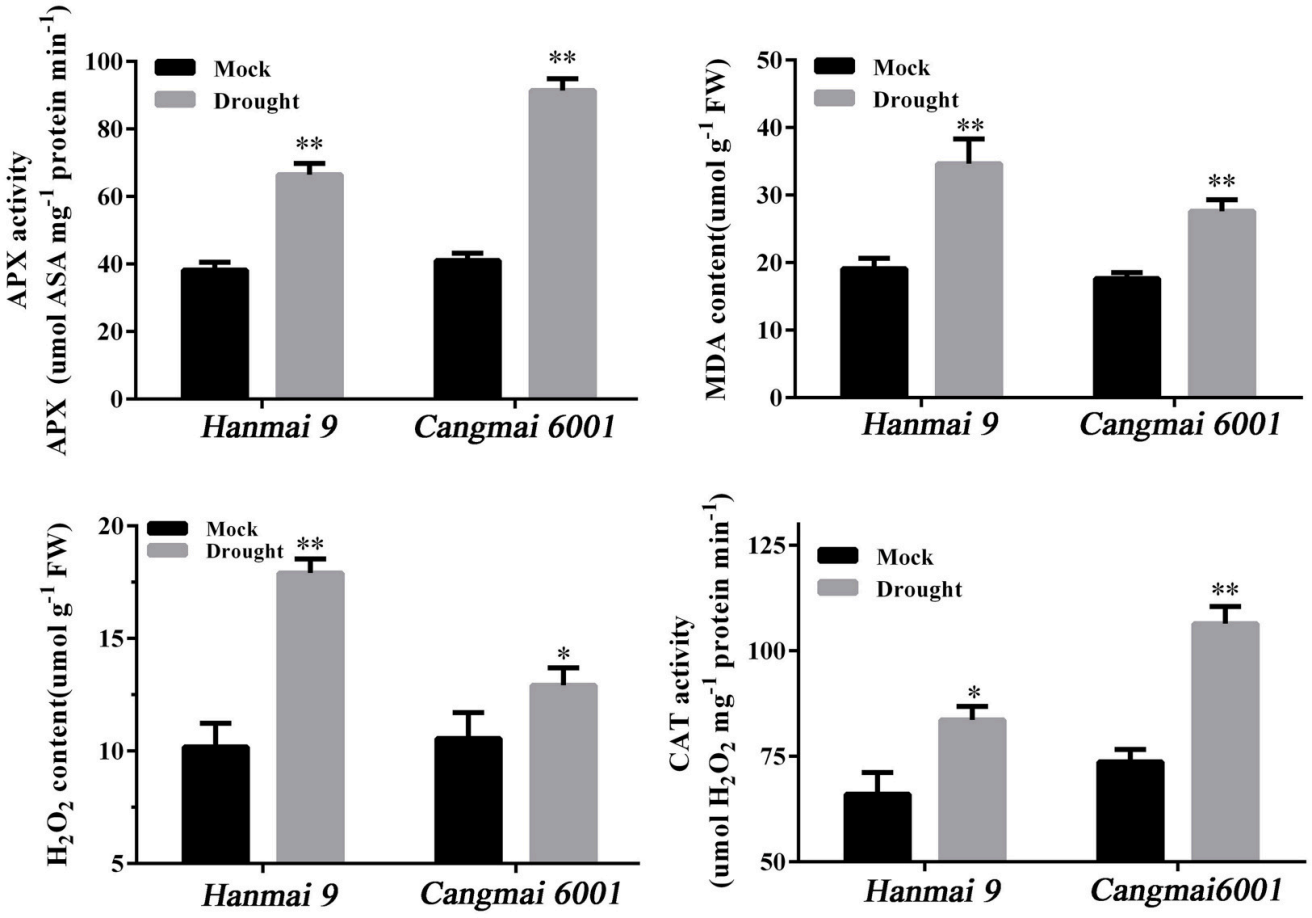
**Drought-related physiological traits in Hanmai 9 and Cangmai 6001**. Data are mean ± standard deviation of three trials. ^*^p < 0.05; ^**^p < 0.01.

The two varieties used for RT-qPCR validation responded differently to drought in terms of drought-related physiological indexes. Cangmai 6001 had higher APX and CAT activities and lower MDA and H_2_O_2_ contents than Hanmai 9 in response to drought stress, thus confirming the better drought tolerance of Cangmai 6001 (Figure 7).

**Figure 8:**
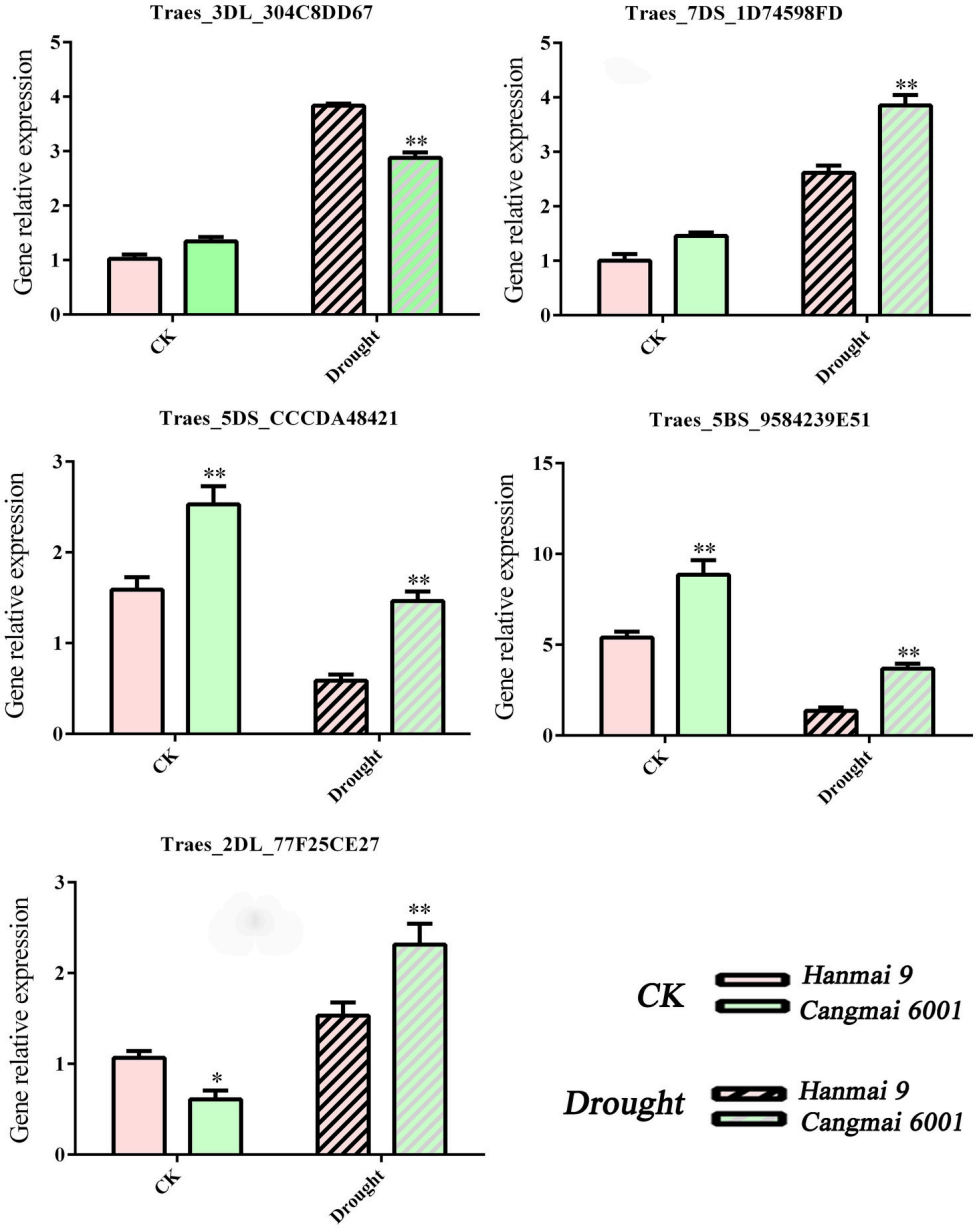
**RT-qPCR validation of selected genes in Hanmai 9 and Cangmai 6001**. Data are mean ± standard deviation of three trials. ^*^p < 0.05; ^**^p < 0.01.

The RT-qPCR confirmed the involvement of five selected DEGs in drought tolerance under different genetic backgrounds. For example, the up-regulated DEG Traes_3DL_304C8DD67 (encoding VPE) shared by T4 and T5 increased in Cangmai 6001 and Hanmai 9, but more so in the drought-sensitive Hanmai 9. The two down-regulated CC-type GRXs genes (Traes_5DS_CCCDA48421 and Traes_5BS_9584239E51) declined less in Cangmai 6001 than Hanmai 9, while Traes_2DL_77F25CE27 and Traes_7DS_1D74598FD increased more in Cangmai 6001 than Hanmai 9 under drought, which were in line with RNA-seq results (Figure 8).

## Discussion

### Plant response to drought stress applied during different growth periods

The total dry biomass of plants in blocks A and C declined, indicating a negative impact of drought on wheat growth and development, compared with the control block D. Drought had the greatest effect on grain number related traits of plants in block A, with reductions of 6.45% in spike number, 11.02% in spikelet number per spike and 6.64% in grain setting rate, compared with the control block D. Plants in block C had the lowest thousand grain weight, declining by 6.24% compared with control block D (Table 1). It can be concluded from these observations that drought during early reproductive periods (before flowering) mainly impacted grain number related traits whereas drought during flowering affected grain weight. This is consistent with other studies on wheat, where drought during stem extension caused floret and whole spikelet death and drought during grain filling reduced grain size and weight (Oosterhuis and Cartwright, 1983; Dorion et al., 1996; Ji et al., 2010; Pradhan et al., 2012).

A critical and direct parameter for measuring drought damage is grain yield. It is well established that drought during flowering and grain filling can severely reduce wheat yield (Farooq et al., 2014). Some studies have focused on the drought-responsive genes during these periods (Aprile et al., 2009, 2013). In contrast, genes responsive to drought at early reproductive periods were mostly unknown. A study conducted by Ugarte et al. (2007) indicated that the high temperature during stem elongation reduced wheat yield by 46%, compared with that from booting to anthesis (27%) and heading to anthesis (15%) (Ugarte et al., 2007) Our results with drought stress followed a similar trend, where grain yield in block A (drought stress applied during early reproductive stages before flowering) declined by 6.1% compared with 2.4% in block C (drought stress applied after flowering periods) (Table 1). Therefore, like heat, drought stress during early reproductive periods may affect wheat yields more than drought during flowering periods. Investigation of genes responsive to drought during the early reproductive stage is needed.

### Effect of drought stress on gene expression at early reproductive stages

In contrast to previous wheat transcriptome studies where hundreds or even thousands of DEGs have been identified (Aprile et al., 2009, 2013; Li et al., 2012; Liu et al., 2015), only 309 DEGs were identified in our study. The likely reason for this is our experimental design (irrigation strategy). The small difference in the level of drought stress between control and test samples can reduce the number of DEGs identified in transcriptome analyses (Aprile et al., 2009). We adopted a two-times irrigation schedule in the control block as this schedule is widely adopted in the North China Plain, where about two-thirds of China's total wheat output is produced (Lu and Fan, 2013). This two-times irrigation strategy is sufficient to maintain wheat production under drought stress conditions (Li et al., 2005; Yang et al., 2006). By simulating this strategy, we aimed to study the molecular mechanisms to maintain wheat production in local areas and to provide useful genetic resources for drought tolerance improvement.

In our study, the most DEGs were identified at T4 (pistil and stamen differentiation stage). The chalcone synthase gene (CHS) and flavonoid 3′-hydroxylase gene (F3H) in the enriched flavonoid biosynthesis pathway, and fructan biosynthesis genes (1-FFT, 6-SFT, and 1-SST) in the enriched starch and sucrose metabolism pathways were known to be associated with dorught tolerance in wheat (Kawakami and Yoshida, 2002; Ithal and Reddy, 2004; Ji et al., 2010; Ma et al., 2014; Wang et al., 2016). The two enriched GO terms at this stage (protein disulfide oxidoreductase activity and cell redox homeostasis) were associated with the same group of DEGs, including one up-regulated and seven down-regulated DEGs. These DEGs were annotated to CC-type glutaredoxins (GRXs), which are small (10–15 kDa) oxidoreductases that catalyze the reduction of disulfide bonds of their substrate proteins in the presence of glutathione (GSH). The loss-of-function mutants of CC-type GRXs genes in *Arabidopsis*, maize and rice had fewer petal primordia, disrupted anther lobe differentiation, and disabled meiotic entry of sporogenous cell progenies (leading to male sterility; Xing and Zachgo, 2008; Wang et al., 2009; Hong et al., 2012; Kelliher and Walbot, 2012). Therefore, down-regulation of CC-type GRXs genes in wheat may explain the spikelet death caused by drought during the early reproductive stage (Oosterhuis and Cartwright, 1983), but this needs further investigation. Meanwhile, a previous study reported increased sensitivity to oxidative stress in *Arabidopsis* mutants with knock down GRX genes (Li et al., 2014). Under drought stress, the two CC-type GRXs genes (Traes_5DS_CCCDA48421 and Traes_5BS_9584239E51) declined less in Cangmai 6001 than in Hanmai 9, which was in line with its drought tolerance (Figures 7, 8).

The subcellular location of the proteins of the DEGs at T4 indicated that the peroxisome, nucleus and mitochondria might be sensitive to drought stress damage. This finding differed from those of *Hippophae rhamnoides* seedlings, where the proteins responsive to drought stress were mainly located in the chloroplast, mitochondria and secretory pathway (He et al., 2016). Plant oxidative stress can be caused by excess H_2_O_2_ accumulation under drought stress, which severely damages biomolecules due to the elevated and non-metabolized cellular H_2_O_2_ (Sofo et al., 2015). In this study, Hanmai 9 accumulated significantly more H_2_O_2_ under drought stress than Cangmai 6001, and significantly less H_2_O_2_-metabolizing enzymes such as CAT and APX. Peroxisomes are involved in the response of plants to biotic and abiotic stresses (Kaur et al., 2009; Hu et al., 2012; Smith and Aitchison, 2013). Importantly, plant peroxisomes are involved in conserved functions (e.g., detoxification of reactive oxygen species, or ROS) and plant-specific functions (e.g., photorespiration and metabolism of hormones) (Kaur et al., 2009; Hu et al., 2012; Smith and Aitchison, 2013). The DEGs encoding proteins in the peroxisome may be involved directly or indirectly in ROS scavenging in wheat at early developmental stages under drought.

At the anther differentiation stage (T5), the genes involved in photosynthetic activities were significantly affected by drought. The enriched GO terms (ribulose-bisphosphate carboxylase activity and carbon fixation) contained three up-regulated DEGs encoding ribulose-1,5-bisphosphate carboxylase/oxygenase (Rubisco). Rubisco catalyzes the first step in net photosynthetic CO_2_ assimilation and is a central component of photosynthesis—a new “green revolution” (Whitney et al., 2011; Long et al., 2015). Studies have shown that Rubisco activase acclimates *in situ* to high temperature when the stress is imposed at a slow rate in the field. Altered expression of Rubisco activase might be crucial for continued CO_2_ fixation under drought stress, protecting plant photosynthetic capacity (Crafts et al., 1998; Law and Crafts-Brandner, 2001). In addition, Pelloux et al. (2001) and Fu et al. (2007) reported that the expression pattern of some Rubisco genes under drought stress had a temporal manner in Aleppo pine and rice, which increased at an early stage of growth but then decreased at a later stage. Similarly, of the three wheat Rubisco genes in our study, one (Traes_7DS_1D74598FD) was up-regulated by drought at T5 and later down-regulated at T8. Further experiments are required to investigate the mechanism for different expression of Rubisco genes during wheat growth.

At tetrad stage (T6), eight DEGs were identified. Most of these DEGs had unknown functions in wheat. However, the stomatal movement related gene Traes_2DS_BB50DEEF8 (encoding CHLH) which was shared between T6 and T4 arose our interests. As plants lose over 95% of their water via transpiration through stomata, the engineering of stomatal activity is believed to be a promising approach to reduce the water requirement of crops and to enhance productivity under stress conditions (Schroeder et al., 2001).

### Effect of drought stress on gene expression during flowering

Compared with early reproductive stages, drought stress during flowering had less effect on wheat gene expression in the developing grains because fewer DEGs were detected during early flowering (T8; 6 DEGs) and grain filling (T9; 25 DEGs). We found that drought stress induced several grain development-related genes. For example, during early flowering, the DEG TRAES3BF077200010CFD_g was annotated to the lipid-transfer protein LTPL38, which may contribute to mineral accumulation in wheat grains (Singh et al., 2014). At grain filling (T9), four DEGs in the enriched KEGG pathway “starch and sucrose metabolism” stood out: three were annotated to the wheat *a-Amy3* gene that encodes a-amylase, and one was annotated to a rice gene encoding glycoside hydrolase family 31 proteins, a-glucosidase (ONG2) (Nakai et al., 2007). Both a-amylase and ONG2 can hydrolyze raw starch granules (Sissons and Macgregor, 1994). a-Amy3 mainly provides energy for early developing wheat grains in the spikes (Zanetti et al., 2000). Rice ONG2 and its mRNA is only produced during ripening and remains an active and key enzyme in starch degradation metabolism during the initial stage of germination (Sissons and Macgregor, 1994; Nakai et al., 2007). The change in these genes under drought may affect wheat grain development; we will investigate the functions of these genes in future experiments.

## Author contributions

Conceived and designed the experiments: JM, RL, and GY. Performed the experiments: RL, HW, and DL. Analyzed the results: JM, RL, and YL. Contributed reagents/materials: XW, YZ, WZ, and HD. Contributed to the writing of the manuscript: JM, RL, and GY.

### Conflict of interest statement

The authors declare that the research was conducted in the absence of any commercial or financial relationships that could be construed as a potential conflict of interest.
