# Supplementary material for: Transcriptomics Analyses Reveal Wheat Responses to Drought Stress during Reproductive Stages under Field Conditions
Source: Front Plant Sci. 2017 Apr 21;8:592. doi: 10.3389/fpls.2017.00592 (PMC5399029; doi:10.3389/fpls.2017.00592)
Supplement: Supplementary file 6 [file DataSheet1.docx]

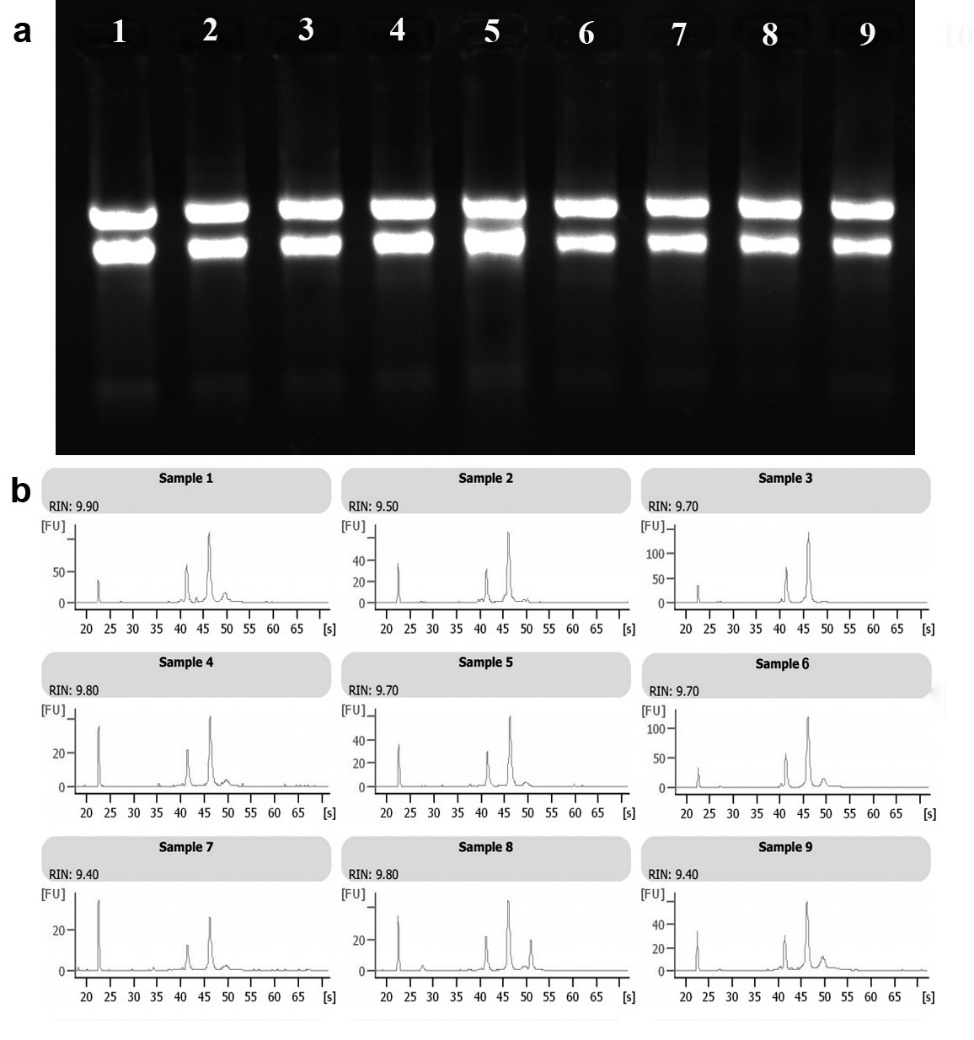


Fig. S1 RNA quality in the RNAseq experiment (partial). a, Determination of RNA quality by agarose gel electrophoresis. b, RNA quality was detected by the Agilent 2100 bioanalyzer.
