# Supplementary material for: Transcriptomics Analyses Reveal Wheat Responses to Drought Stress during Reproductive Stages under Field Conditions
Source: Front Plant Sci. 2017 Apr 21;8:592. doi: 10.3389/fpls.2017.00592 (PMC5399029; doi:10.3389/fpls.2017.00592)
Supplement: Supplementary file 7 [file DataSheet2.docx]

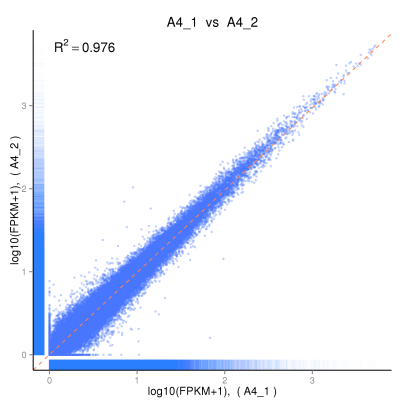

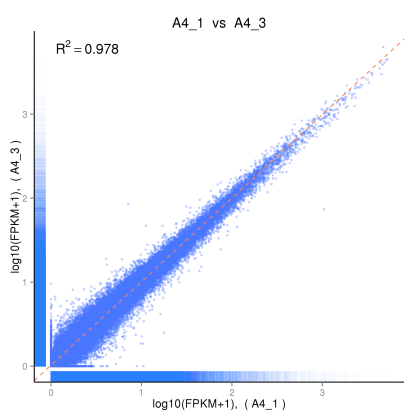

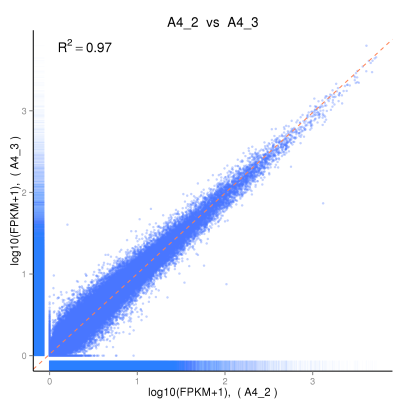


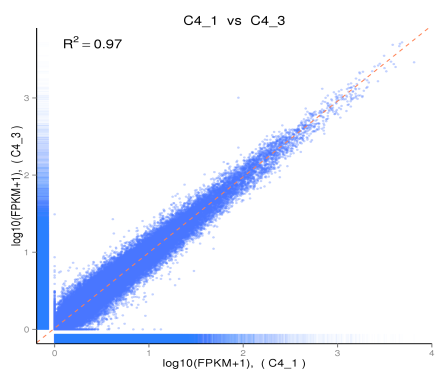

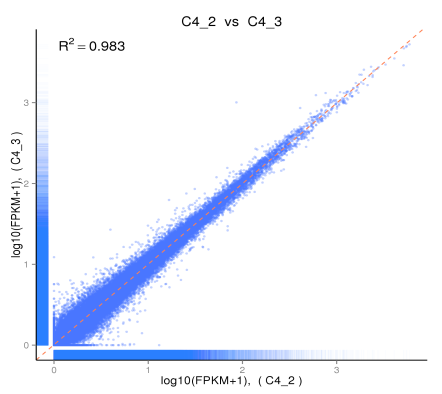

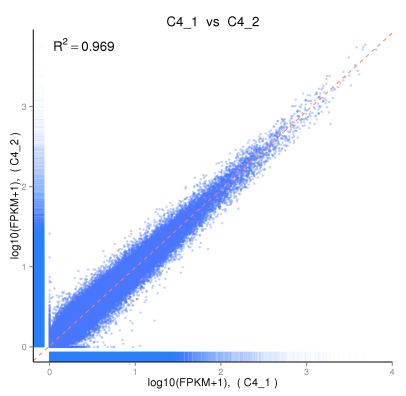


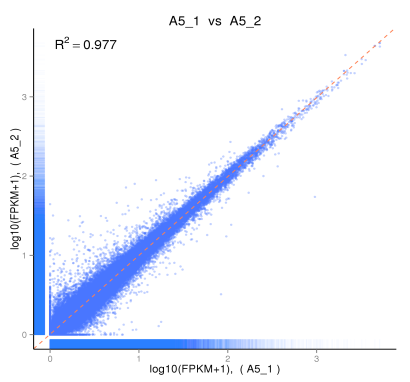

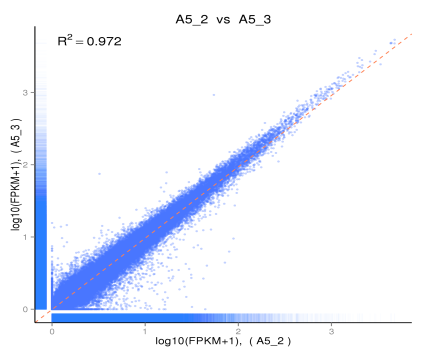

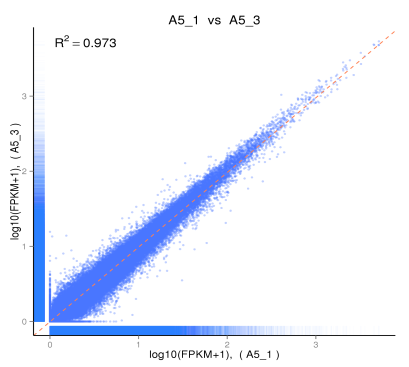


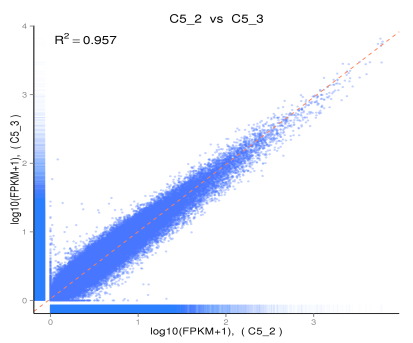

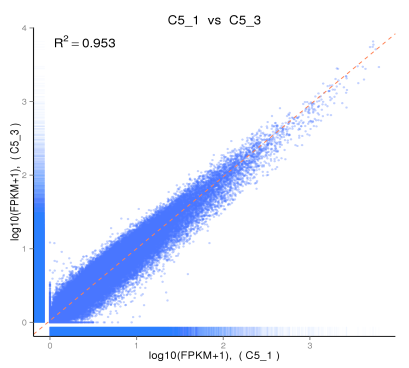

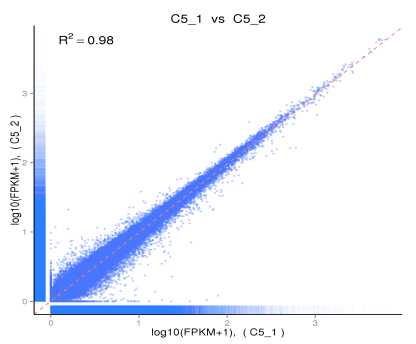


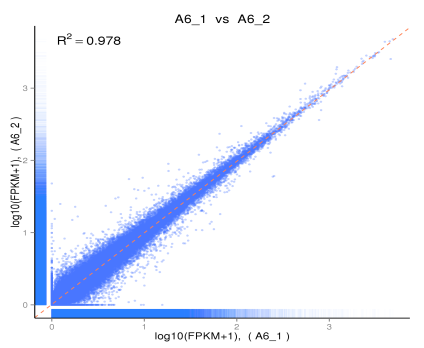

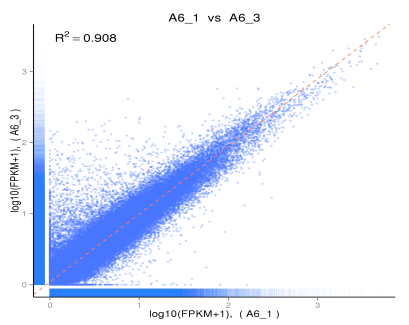

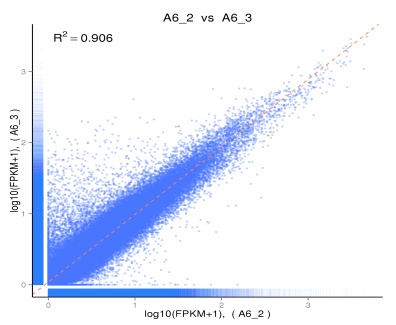

Fig. S2 The Pearson's correlation coefficients between three replicates in each block at five time points (T4, T5, T6, T8 and T9) were between 0.82 to 0.98.
